# Radiation therapy of breast cancer in the Nahe Breast Center: first results of an analysis in the context of health services research

**DOI:** 10.1007/s00066-023-02157-8

**Published:** 2023-11-10

**Authors:** Ralph Mücke, Gabor Heim, Robert Gosenheimer, Volker Schmitz, Christoph Schulz, Per Knoeß, Khashayar Fakhrian, Christina Harvey, Christiane Mücke, Gabriele Lochhas, Ute Metzmann, Matthias Bussmann, Markus Paschold

**Affiliations:** 1 Radiotherapy RheinMainNahe, Mainz-Ruesselsheim-Bad Kreuznach, Mühlenstraße 39a, 55543 Bad Kreuznach, Germany; 2 Department of Radiotherapy and Radiation Oncology, Marien Hospital Herne, Ruhr University Bochum, Bochum, Germany; 3 Department of Gynecology, Hospital Sankt Marienwoerth, Bad Kreuznach, Germany; 4 Department of Internal Medicine, Hospital Sankt Marienwoerth, Bad Kreuznach, Germany; 5 Oncological Practice, Bad Kreuznach, Germany; 6 Institute of Pathology, Bad Kreuznach, Germany; 7 Radiotherapy, Aschaffenburg, Germany; 8 Medical Management Board, Hospital Sankt Marienwoerth, Bad Kreuznach, Germany; 9 Department of Surgery, Hospital Sankt Marienwoerth, Bad Kreuznach, Germany

**Keywords:** Overall survival, Local recurrence, Endocrine therapy, Chemotherapy, Distant metastasis

## Abstract

**Background:**

The first evaluation of radiotherapy results in patients with breast cancer treated as part of a multimodal oncologic therapy in the Nahe Breast Center is presented. Analysis of the results was performed using an in-practice registry.

**Patients and methods:**

From September 2016 to December 2017, 138 patients (median age 62.5 years; range 36–94 years) with breast cancer (right side, *n* = 67; left side, *n* = 71) received adjuvant radiation therapy. Of these, 103 patients received gyneco-oncologic care at the Nahe Breast Center, and 35 were referred from outside breast centers. The distribution into stages was as follows: stage I, *n* = 48; stage II, *n* = 68; stage III, *n* = 19; stage IV, *n* = 3. Neoadjuvant chemotherapy was given to 19 and adjuvant chemotherapy to 50 patients. Endocrine treatment was given to 120 patients. Both 3D conformal (*n* = 103) and intensity-modulated (*n* = 35) radiotherapy were performed with a modern linear accelerator.

**Results:**

With a median follow-up of 60 months (1–67), local recurrence occurred in 4/138 (2.9%) and distant metastasis in 8/138 (5.8%) patients; 7/138 (5.1%) patients died of their tumors during the follow-up period. The actuarial 5‑year local recurrence-free survival of all patients was 97.1%, and the actuarial 5‑year overall survival of all patients was 94.9%. We observed no grade 3 or 4 radiogenic side effects.

**Conclusion:**

The results of radiotherapy for breast carcinoma at the Nahe Breast Center are comparable to published national and international results. In particular, the local recurrence rates in our study, determined absolutely and actuarially, are excellent, and demonstrate the usefulness of radiotherapy.

## Introduction

This study is an initial evaluation of adjuvant radiation as part of the multimodal therapy of patients with breast cancer treated at the Nahe Breast Center in Bad Kreuznach. The study was performed to evaluate the quality of the treatment internally and to compare it with the results of national and international studies. The analysis is considered very important in identifying any deviations so they can be corrected if necessary. Data were collected using an internal practice registry in radiotherapy, which was kept in a disciplined manner over the years for all patients. Parameters such as type of surgery and systemic therapy were included. To ensure a sufficient follow-up period, only patients who received radiation from September 2016 to the end of 2017 were included in the analysis.

## Patients and methods

From September 2016 to December 2017, 138 patients (median age 62.5 years; range 36–94 years) with breast cancer (right side, *n* = 67; left side, *n* = 71) received adjuvant radiation therapy. Of these, 103 patients received gyneco-oncologic care at the Nahe Breast Center, and 35 were referred from outside breast centers. The distribution into stages was as follows: stage I, *n* = 48; stage II, *n* = 68; stage III, *n* = 19; stage IV, *n* = 3. Neoadjuvant chemotherapy was given to 19 and adjuvant chemotherapy to 50 patients. Endocrine treatment was given to 120 patients.

Radiation therapy was performed with a modern linear accelerator from Elekta (Stockholm, Sweden). All modern radiation techniques such as 3D conformal radiation (*n* = 103) and intensity-modulated radiation (IMRT; *n* = 35) were used. Radiation therapy target volumes and total doses were determined in each case according to the national S3 guideline, with each individual case discussed in an interdisciplinary tumor conference prior to the start of radiation. The median total radiation dose after breast-conserving surgery (*n* = 107) was 62 Gy (50–66), and after mastectomy (*n* = 31) it was 50 Gy (40–60).

The distribution of patient and radiation criteria is given in Tables 1 and 2.

**Table 1:** Patient data

T‑stage	T0	1/138	(0.7%)
	T1	75/138	(54.4%)
	T2	45/138	(32.6%)
	T3	13/138	(9.4%)
	T4	4/138	(2.9%)
N‑stage	N0	91/138	(65.9%)
	N1	31/138	(22.5%)
	N2	12/138	(8.7%)
	N3	4/138	(2.9%)
Grading	G1	24/138	(17.4%)
	G2	79/138	(57.2%)
	G3	35/138	(25.4%)
Receptor status	ER+/PR+	120/138	(87.0%)
	ER−/PR−	18/138	(13.0%)
HER-2/neu status	Negative	126/138	(91.4%)
	Positive	12/138	(8.6%)
Lymph node surgery	Sentinel Node	98/138	(71.0%)
	Axilla-Dissection	40/138	(29.0%)
Surgery	Breast Conserving	107/138	(77.5%)
	Ablatio	31/138	(22.5%)
Resection status	R0	138/138	(100.0%)

*T-stage* tumor size, *N-stage* lymph node involvement, *HER-2/neu status* human epidermal growth factor receptor type 2

**Table 2:** Radiation data

Fractionation	Hypofractionated	4/138	(2.9%)
	Normofractionated	134/138	(97.1%)
RT technique	3D	103/138	(74.6%)
	IMRT	35/138	(26.4%)
Boost	Simultaneous	85/138	(61.6%)
	Sequential	17/138	(12.3%)
	No Boost	36/138	(26.1%)
RT of lymph nodes	Yes	48/138	(34.8%)
	No	90/138	(65.2%)

*IMRT* intensity-modulated radiation therapy, *3D* three-dimensional conformal radiation therapy, *RT* radiation therapy

### Statistics

All data were stored and analyzed using the SPSS statistical package 29.0 (IBM. Corp., Armonk, NY, USA). Descriptive statistics were computed for continuous and categorical variables. The statistics computed included median and interquartile ranges of ordinal variables, means and standard deviations of continuous variables, and frequencies and relative frequencies of categorical factors. The period to death or recurrence or last follow-up was estimated with the Kaplan–Meier method. Differences between curves were assessed by the Mantel log-rank test for censored survival data. All *p*-values were from two-sided statistical tests, and values of *p* < 0.05 were considered statistically significant.

## Results

With a median follow-up of 60 months (1–67), local recurrence occurred in 4/138 (2.9%) and distant metastasis in 8/138 (5.8%) patients; 7/138 (5.1%) patients died of their tumors during the follow-up period.

### Local recurrence-free survival

**Fig. 1:**
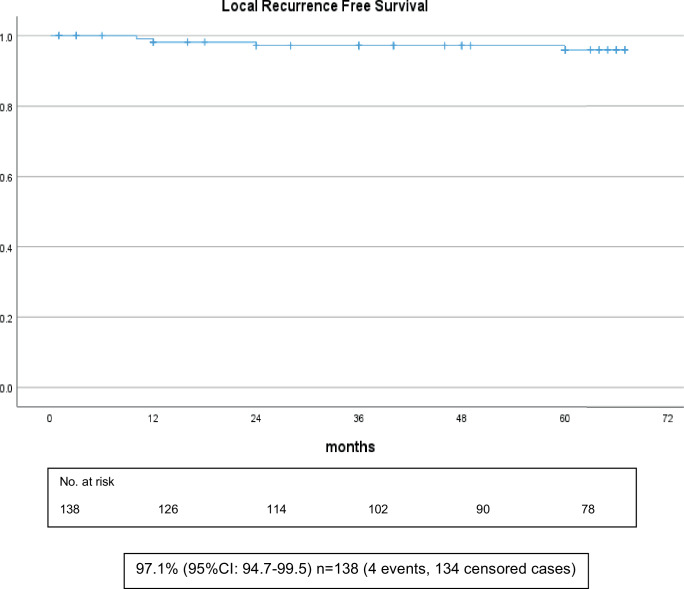
Local recurrence-free survival of all patients (*n* = 138)

The actuarial 5‑year local recurrence-free survival of all patients was 97.1% (Fig. 1).

### Overall survival

**Fig. 2:**
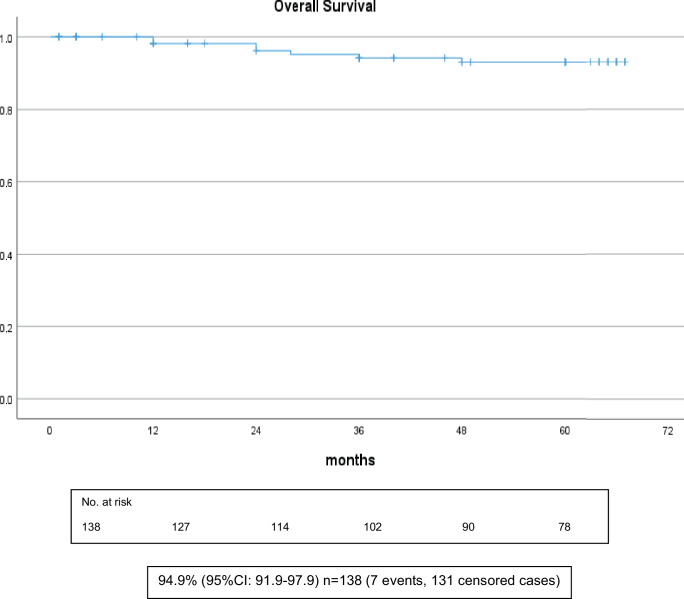
Overall survival of all patients (*n* = 138)

The actuarial 5‑year overall survival of all patients was 94.9% (Fig. 2). Further calculated survival data are shown in Tables 3 and 4.

**Table 3:** Local recurrence-free survival data depending on endocrine therapy, chemotherapy, and grading

With endocrine therapy	*n* = 120	1 event, 119 censored cases	99.2% (95% CI: 97.8–100.6)	*p* < 0.001
Without endocrine therapy	*n* = 18	3 events, 15 censored cases	83.3% (95% CI: 69.8–96.8)	
Without chemotherapy	*n* = 69	0 events, 69 censored cases	100.0%^a^	*p* < 0.001
With adjuvant chemotherapy	*n* = 50	1 event, 49 censored cases	98.0% (95% CI: 94.7–101.3)	
With neoadjuvant chemotherapy	*n* = 19	3 events, 16 censored cases	84.2% (95% CI: 69.5–98.9)	
Grade 1	*n* = 24	0 events, 24 censored cases	100.0%^a^	*p* < 0.001
Grade 2	*n* = 79	0 events, 79 censored cases	100.0%^a^	
Grade 3	*n* = 35	4 events, 31 censored cases	88.6% (95% CI: 77.7–98.6)	

*CI *confidence intervals

^a^No CI are calculated because all cases are censored

**Table 4:** Overall survival data depending on endocrine therapy, chemotherapy, and grading

With endocrine therapy	*n* = 120	5 events, 115 censored cases	95.8% (95% CI: 92.8–98.8)	*p* = 0.201
Without endocrine therapy	*n* = 18	2 events, 16 censored cases	88.9% (95% CI: 77.7–100)	
Without chemotherapy	*n* = 69	0 events, 69 censored cases	100.0%^a^	*p* = 0.019
With adjuvant chemotherapy	*n* = 50	5 events, 45 censored cases	90.0% (95% CI: 84.2–95.8)	
With neoadjuvant chemotherapy	*n* = 19	2 events, 17 censored cases	89.5% (95% CI: 73.1–105.9)	
Grade 1	*n* = 24	0 events, 24 censored cases	100.0%^a^	*p* < 0.039
Grade 2	*n* = 79	3 events, 76 censored cases	96.2% (95% CI: 93.0–99.4)	
Grade 3	*n* = 35	4 events, 31 censored cases	88.6% (95% CI: 78.1–99.1)	

*CI *confidence intervals

^a^No CI are calculated because all cases are censored

Toxicity data are shown in Tables 5 and 6.

**Table 5:** Toxicity data at the end of radiotherapy

Radiation dermatitis	Grade 0	24/138	(17.4%)
	Grade 1	100/138	(72.5%)
	Grade 2	14/138	(10.1%)
	Grade 3	0/138	(0%)
	Grade 4	0/138	(0%)
Breast pain	Grade 0	125/138	(90.6%)
	Grade 1	9/138	(6.5%)
	Grade 2	4/138	(2.9%)
	Grade 3	0/138	(0%)
	Grade 4	0/138	(0%)
Fatigue	Grade 0	59/138	(42.7%)
	Grade 1	71/138	(51.5%)
	Grade 2	8/138	(5.8%)
	Grade 3	0/138	(0%)
	Grade 4	0/138	(0%)
Pneumonitis	Grade 0	135/138	(97.8%)
	Grade 1	3/138	(2.2%)
	Grade 2	0/138	(0%)
	Grade 3	0/138	(0%)
	Grade 4	0/138	(0%)

**Table 6:** Toxicity data during follow-up

Fibrosis	Grade 0	113/138	(81.9%)
	Grade 1	21/138	(15.2%)
	Grade 2	4/138	(2.9%)
	Grade 3	0/116	(0%)
	Grade 4	0/116	(0%)
Hyperpigmentation	Grade 0	98/138	(71.0%)
	Grade 1	34/138	(24.6%)
	Grade 2	6/138	(4.4%)
	Grade 3	0/138	(0%)
	Grade 4	0/138	(0%)
Lymphedema breast	Grade 0	120/138	(87.0%)
	Grade 1	12/138	(8.7%)
	Grade 2	6/138	(4.3%)
	Grade 3	0/138	(0%)
	Grade 4	0/138	(0%)
Lymphedema arm	Grade 0	128/138	(92.7%)
	Grade 1	7/138	(5.1%)
	Grade 2	3/138	(2.2%)
	Grade 3	0/138	(0%)
	Grade 4	0/138	(0%)
Lung fibrosis	Grade 0	135/138	(97.8%)
	Grade 1	3/138	(2.2%)
	Grade 2	0/138	(0%)
	Grade 3	0/138	(0%)
	Grade 4	0/138	(0%)

## Discussion

Our results of breast cancer irradiation in the context of multimodal therapy are in line with nationally and internationally published results. In particular, the 5‑year local recurrence rate for all patients in our study, determined absolutely (2.9%) and actuarially (97.1%), is excellent and demonstrates the usefulness of radiotherapy. It also shows the high professional and technical quality of all employees in our practice. The results confirm that postoperative radiation is the most important and effective measure to reduce the risk of local recurrence [1–12]. If we follow the current literature, this finding applies to all breast cancer subgroups. Thus, all women should be presented for radio-oncology after breast-conserving surgery, and predictive markers for decision-making cannot be relied on.

The currently published PRIME II trial also demonstrates these excellent local effects of radiation, although the authors recommend against radiation for women older than 65 years with hormone receptor positivity after breast-conserving surgery if they consistently take antiestrogenic medication for 5 years. In this study, the local recurrence rate of this group of patients after 10 years without radiation was 8.6%, and with radiation it was 1% [13].

Meta-analyses have even shown that local irradiation not only reduces the local recurrence rate but also reduces the rate of distant recurrences [1, 2].

With the use of state-of-the-art irradiation techniques, as we apply them in our center, both pronounced acute and late radiogenic side effects are very rare: no grade 3 and 4 toxicities occurred in our evaluation, and the pneumonitis rate is only 2.2% (Table 5).

Skin toxicity is comparable to currently published data. Pulmonary toxicity is slightly better, but only grade 1 symptomatic pneumonitis was reported in our study. Asymptomatic pneumonitis was not analyzed [14, 15].

A limitation of this study is the still very small number of patients, especially in the subgroups. We are very aware that this analysis is not a prospective randomized trial. The worse outcomes of patients with chemotherapy and with histological grade 3 disease indicate that these groups included patients with more risk factors. For example, only 4/24 patients (16.6%) with a grading of 1 but 33/35 patients (94.3%) with a grading of 3 received chemotherapy. Regarding nodal status, there were 31/91 patients (34.1%) with negative nodal status and 38/47 patients (80.9%) with positive nodal status who received chemotherapy. We will not further discuss the presented subgroups, such as those who did not receive endocrine treatment and did receive chemotherapy and patients with different grades of disease, but we wanted to show them. Nevertheless, we wanted to check the quality of our work for the first time after a follow-up of 5 years.

The results indicate high professional quality in the treatment of breast cancer at the Nahe Breast Center. Through regular training and quality improvement measures, everything is being done to maintain or even improve this high level of quality.

## Conclusion

The results of radiotherapy for breast cancer at the Nahe Breast Center are comparable to published national and international results. In particular, the local recurrence rates in our study, determined absolutely and actuarially, are excellent and demonstrate the usefulness of radiotherapy.
